# Chondrosarcoma metastasis in the thyroid gland: a case report

**DOI:** 10.1186/1752-1947-8-157

**Published:** 2014-05-20

**Authors:** Youssef Darouassi, Mohamed Mliha Touati, Mehdi Chihani, Karim Nadour, Mostapha Boussouga, Haddou Ammar, Brahim Bouaity

**Affiliations:** 1 ENT department, Avicenne Military Hospital, Marrakech, Morocco; 2 Department of Orthopaedic Surgery, Mohamed V Military Hospital, Rabat, Morocco

**Keywords:** Chondrosarcoma, Metastasis, Thyroid gland

## Abstract

**Introduction:**

Chondrosarcoma metastases in the thyroid gland are exceptional. To the best of our knowledge, only two cases have been previously reported in the literature. Here we report the third case.

**Case presentation:**

We report the case of a 51-year-old Arab woman who presented in 2011 with a diaphyseal chondrosarcoma of her right tibia treated by surgery. In the last quarter of 2013, she presented a hard mass in her thyroid gland with dyspnea and a right laryngeal paresis. She underwent a debulking surgery with tracheostomy in order to prevent difficulty in respiration. The final pathology revealed the diagnosis of a chondrosarcoma metastasis within her thyroid gland. She died several days later.

**Conclusions:**

Even if primary and metastatic chondrosarcomas of the thyroid gland are exceptional, they should be considered in the differential diagnosis of thyroid gland masses. The prognosis is poor but surgery may help preserve quality of life.

## Introduction

Chondrosarcomas are slow-growing invasive tumors. They represent approximately 11% of all primary malignant bone tumors. Only 1 to 12% of chondrosarcomas occur in the head and neck region representing 0.1% of its neoplasms [1]. Metastases in the thyroid gland represent 0.4% of thyroid gland cancers and 0.05% of the patients who underwent thyroid gland surgery had metastases in their thyroid gland, but the number of cases is gradually increasing [2]. Thyroid metastases arising from sarcomas are extremely rare [3]. To the best of our knowledge, only six cases of primary thyroidal chondrosarcomas [4-9] and two cases of chondrosarcoma metastases in the thyroid gland [10,11] have been reported in the literature. The authors report here the third case.

## Case presentation

We report the case of a 51-year-old Arab woman with no medical history; she presented in 2011 with a diaphyseal chondrosarcoma of her right tibia. The staging, including a cervico-thoraco-abdominal computed tomography scan, did not find any distant metastasis. She underwent conservative treatment of her leg with histologically clear margins. After consultation with multidisciplinary staff, and because chondrosarcomas are reputed to be radioresistant and chemoresistant, she did not undergo chemotherapy or radiotherapy. A year later, she received a diaphyseal prosthesis.

**Figure 1:**
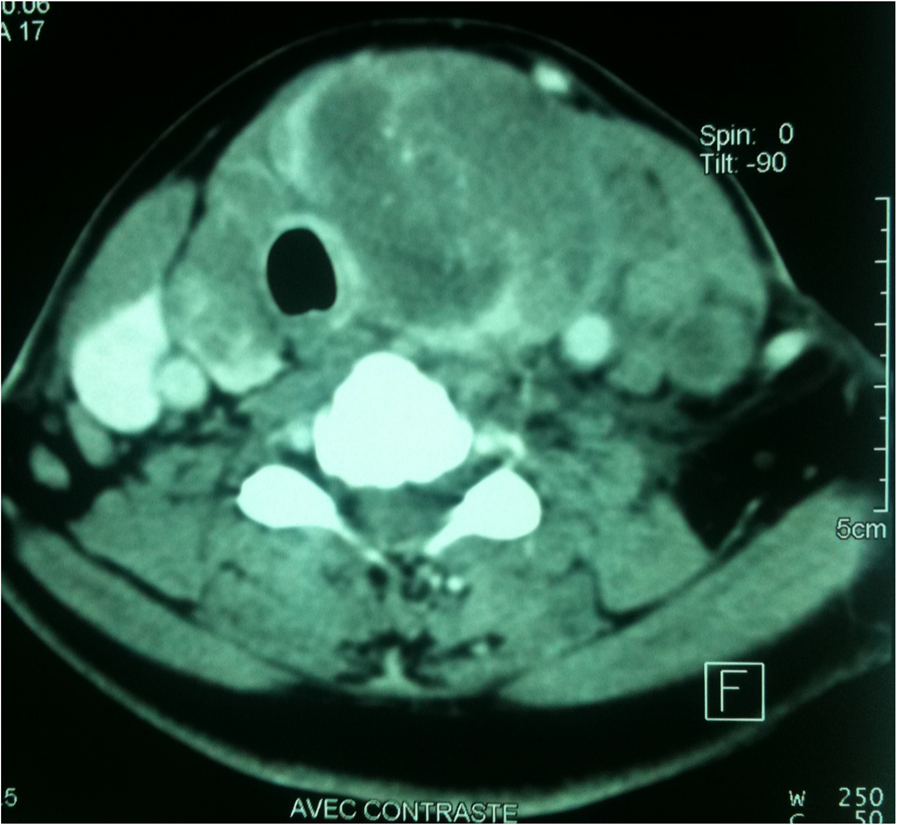
Computed tomography scan showing the thyroid tumor.

**Figure 2:**
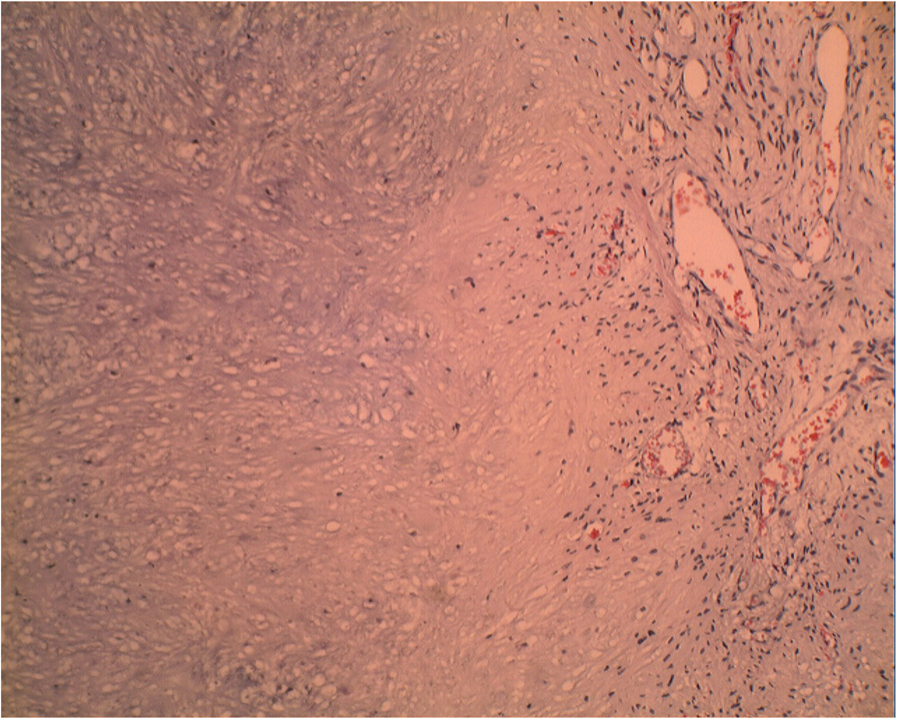
Malignant tumoral proliferation poorly differentiated with chondroid foci (hematoxylin and eosin ×200).

**Figure 3:**
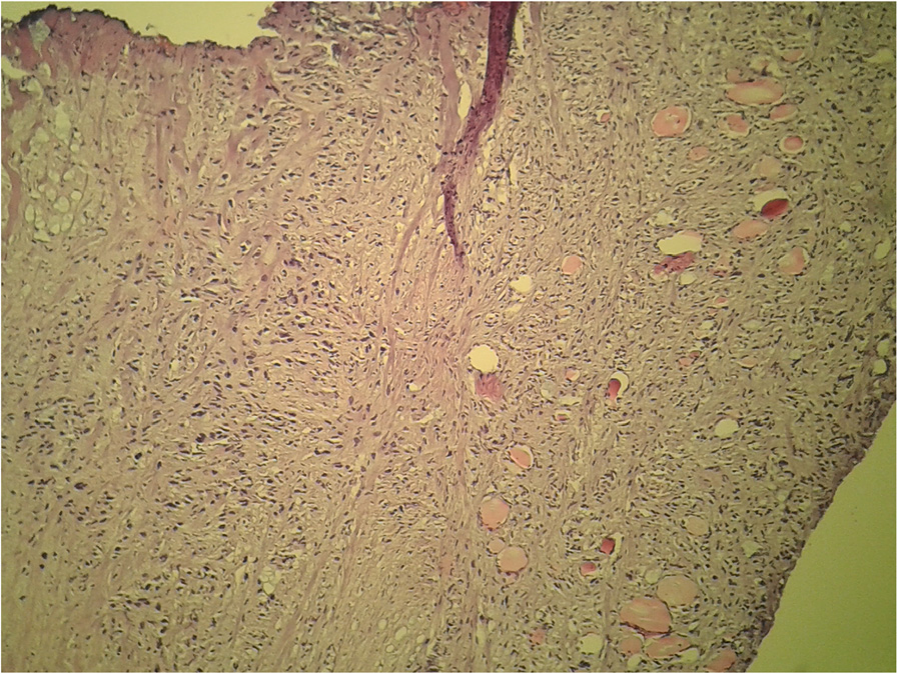
Malignant tumoral proliferation poorly differentiated infiltrating the thyroid gland (hematoxylin and eosin ×200).

In the last quarter of 2013, she presented a quickly growing cervical mass in the thyroid area without pain but with compression signs including dyspnea and hoarseness. A clinical examination found a hard mass in her right thyroid lobe and a right laryngeal paresis. Computed tomography showed a voluminous nodule in her right thyroid lobe with compression of her trachea and endoluminal extension (Figure 1). She underwent a debulking surgery with tracheostomy in order to prevent difficulty in respiration. The extemporaneous histological study found an aspect of sarcoma. The final pathology revealed the diagnosis of a dedifferentiated chondrosarcoma metastasis within the thyroid gland (Figures 2 and 3). She was discharged from the hospital 1 week after surgery but died several days later.

## Discussion

Chondrosarcomas usually appear in patients aged between 40 and 80 years, but many cases of younger patients have been reported, with a slight predilection for the male gender [12-14]. Chondrosarcomas of the head and neck may involve the sinonasal region, jaws, larynx or skull base [1,12,14]. The main clinical manifestation is swelling, and nasal obstruction; pain is infrequently reported in this region [14].

Sarcomas as a primary cancer of the thyroid gland are extremely rare, and have been mainly reported as individual case reports [4]. Metastases in the thyroid gland represent less than 1% of thyroid gland cancers [15,16]. The most frequent primary sites are kidney carcinoma (23%), breast carcinoma (16%), lung carcinoma (15%), melanoma (5%) and colon and laryngeal carcinoma (4.5%) [15]. Although the thyroid is richly vascularized, the frequency of intrathyroid metastases is relatively low; several theories exist to explain it. The most appropriate is the metabolic theory retaining carcinostatic action of iodine, thyroid hormones and antitumor factors concentrated by high-speed blood circulation in the thyroid [10]. A palpable thyroid nodule is discovered in 72% of patients with metastasis in the thyroid gland while some patients complain of a rapidly growing mass in the neck, dysphagia, or hoarseness [2].

On histological examination, 90% of cases of chondrosarcoma are conventional; the other variants account for the remaining 10% and include dedifferentiated, clear cell, myxoid and mesenchymal chondrosarcomas. Each of which can occur in the head and neck [1,13,17]. Different studies have shown that histological grading correlates with prognosis; conventional chondrosarcoma is classified into three grades, from grade I to grade III, according to cellular density, nuclear differentiation, and the size of nucleus [13,17]. Dedifferentiated chondrosarcoma, like in our case, is characterized by nodules of low-grade conventional chondrosarcoma, which are sharply demarcated from areas of a high-grade sarcoma usually showing spindled, pleomorphic or osteosarcomatous phenotypes, it characteristically contains an anaplastic component [1,17].

The most effective treatment modality for chondrosarcoma is surgery with wide en-bloc resection with an adequate histologically clear margin, the type of surgery depends on histologic grade, tumor extension and location [1,13]. Irradiation and chemotherapy do not appear to have a significant effect on survival and they should be used for palliative purposes [1,13]. The treatment of metastases in the thyroid gland is controversial because the prognosis is basically poor, depending on the primary sites. Thyroidectomy is invasive and may not be effective in prolonging survival time; however, it may help preserve the quality of life in case of extension to surrounding tissues [2,10].

## Conclusions

Even if primary and metastatic chondrosarcomas of the thyroid gland are exceptional, they should be considered in the differential diagnosis of thyroid gland masses. The prognosis is poor but surgery may help preserve quality of life. There is no clear consensus for therapy because recommendations are based on case reports.

## Consent

Written informed consent was obtained from the patient’s next to kin for publication of this case report and accompanying images. A copy of the written consent is available for review by the Editor-in-Chief of this journal.

## Competing interests

The authors declare that they have no competing interests.

## Authors’ contributions

All authors contributed in treatment of the patient and in writing the manuscript; they read and approved it.
